# Will virtual multidisciplinary team meetings become the norm for musculoskeletal oncology care following the COVID-19 pandemic? - experience from a tertiary sarcoma centre

**DOI:** 10.1186/s12891-020-03925-8

**Published:** 2021-01-05

**Authors:** Raja Bhaskara Rajasekaran, Duncan Whitwell, Thomas D. A. Cosker, Christopher L. M. H. Gibbons, Andrew Carr

**Affiliations:** 1 The Oxford Bone Tumour & Soft Tissue Sarcoma Service, Nuffield Department of Orthopaedics, Rheumatology and Musculoskeletal Sciences, Nuffield Orthopaedic Centre, 17, Horwood Close, Headington, Windmill Road, Oxford, OX3 7LD UK; 2 Botnar Research Centre, Nuffield Department of Orthopaedics, Rheumatology and Musculoskeletal Sciences, University of Oxford, Oxford, OX3 7LD UK

**Keywords:** COVID-19, Multidisciplinary team, Virtual MDT, Sarcoma, Bone tumours

## Abstract

**Background:**

Like with all cancers, multidisciplinary team (MDT) meetings are the norm in bone and soft tissue tumour (BST) management too. Problem in attendance of specialists due to geographical location is the one of the key barriers to effective functioning of MDTs. To overcome this problem, virtual MDTs involving videoconferencing or telemedicine have been proposed, but however this has been seldom used and tested. The COVID-19 pandemic forced the implementation of virtual MDTs in the Oxford sarcoma service in order to maintain normal service provision. We conducted a survey among the participants to evaluate its efficacy.

**Methods:**

An online questionnaire comprising of 24 questions organised into 4 sections was circulated among all participants of the MDT after completion of 8 virtual MDTs. Opinions were sought comparing virtual MDTs to the conventional face-to-face MDTs on various aspects. A total of 36 responses were received and were evaluated.

**Results:**

72.8% were satisfied with the depth of discussion in virtual MDTs and 83.3% felt that the decision-making in diagnosis had not changed following the switch from face-to-face MDTs. About 86% reported to have all essential patient data was available to make decisions and 88.9% were satisfied with the time for discussion of patient issues over virtual platform. Three-fourths of the participants were satisfied (36.1% - highly satisfied; 38.9% - moderately satisfied) with virtual MDTs and 55.6% of them were happy to attend MDTs only by the virtual platform in the future. Regarding future, 77.8% of the participants opined that virtual MDTs would be the future of cancer care and an overwhelming majority (91.7%) felt that the present exercise would serve as a precursor to global MDTs involving specialists from abroad in the future.

**Conclusion:**

Our study shows that the forced switch to virtual MDTs in sarcoma care following the unprecedented COVID-19 pandemic to be a viable and effective alternative to conventional face-to-face MDTs. With effective and efficient software in place, virtual MDTs would also facilitate in forming extended MDTs in seeking opinions on complex cases from specialists abroad and can expand cancer care globally.

**Supplementary Information:**

The online version contains supplementary material available at 10.1186/s12891-020-03925-8.

## Background

Central to cancer management in the modern era is the role of a multi-disciplinary team (MDT) meeting, which optimizes coordination between healthcare professionals in their management decisions of patients. Enabling better coordination and communication, MDTs have facilitated improved decision-making amongst specialists benefitting patient care immensely [1–3]. As the standard of care in most countries, MDTs have positively influenced patient assessment and management practices in cancer care especially with modification of diagnosis, thereby ensuring the appropriate treatment plan is undertaken [4–6].

Following the implementation of the National Cancer Act in 1971 in US by President Richard Nixon, there was increased emphasis on collaboration among different specialities to deliver coordinated care, which initiated MDT-based care in cancer patients [7–9]. By 1989 in the US, over 90% of hospitals with greater than 100 beds and 85% of the hospitals with fewer than 100 beds had a tumour board to deal with cancer care [8]. In the UK, following the recommendations of the Calman-Hine report in 1995, MDTs have become the norm with almost all cancer cases in the UK having their management discussed in an MDT setting [9]. Nowadays, MDTs meetings have been widely implemented in many countries including UK, Australia, and Europe and also in developing ones [1, 9]. Although they were initially employed in the management of common cancers (breast, gastrointestinal, lung and colorectal) [10] they are now being commonly used in rare tumours too such as pancreas, lymphoma and sarcoma [11].

Unlike other cancers, bone and soft tissue tumours (BST) and their MDTs are unique in many ways. Primary bone and soft tissue tumours are a rare form of cancer accounting for less than 1% of all diagnosed malignancies [11]. In 2010, there were 531 new cases of bone sarcoma and 3298 new cases of soft tissue sarcoma in the UK, in stark contrast to nearly 55,200 new cases of breast cancer reported every year. In addition to their low incidence compared to other tumours, they have a broad presentation [12]. They may involve any region of the body and any part of the skeleton, thereby bringing in the role of site-specific surgeons. These tumours also have a bimodal age specific incidence rate with peaks in incidence seen in teenagers and young adults (TYA) and elderly patients. The rarity of these cases, coupled with the complexity in management and presentation, warrants treatment by specialists with training and expertise in musculoskeletal oncology.

There are five reference centres in the UK approved for the management of BST, each with a fully accredited MDT [12]. In a routine BST MDT in the UK, apart from discussing bone and soft tissue malignancies, the meetings are also a platform to triage complex musculoskeletal diseases including infections and ‘tumour mimicking conditions’. Hence, the diagnosis and treatment planning of these conditions in an MDT meeting involves a diverse range of specialists. In our sarcoma service, more than 35 members attend the weekly MDT, chaired by an orthopaedic oncology surgeon and a musculoskeletal radiologist, in order to formulate an appropriate management pathway for all referred patients. The number of active participants in these meetings is an essential element in the decision-making process and, therefore, in the delivery of effective treatment to cancer patients.

One of the key limiting factors to optimal functioning of an MDT is the inconsistent attendance of essential specialists at meetings [13, 14]. Bringing together all clinicians under one roof is a practical problem and non-attendance of key personnel hampers and delays decision making in an MDT. This is a greater problem in BST MDTs where multiple specialists are involved. To overcome this problem, teleconferencing and the use of virtual platforms have been proposed but rarely used to date [1, 15, 16]. Conventional face-to-face MDTs are still practised across cancer networks worldwide and remain the standard of care.

The COVID-19 pandemic has placed an unprecedented strain on healthcare systems globally, including the more time critical services, such as cancer and trauma [17–19]. Essential services must continue unhindered, as any delay in treatment would result in adverse patient outcomes and also increase mortality. Maintaining a routine MDT to triage cases is absolutely imperative. Due to the enforced social distancing measures and the risk of healthcare professionals contracting the disease, the pandemic necessitated MDTs being switched to virtual platforms [20].

We wanted to not only evaluate the efficacy of this exercise among participants, but we also aimed to assess whether this could represent the future of cancer care. We collected the opinions of all participants (core and extended members) of our Sarcoma MDT and surveyed their opinions on MDTs over virtual platforms compared to conventional face-to-face MDTs (Table 1).

**Table 1:** List of healthcare professionals routinely attending the MDT at our Sarcoma Service

Core Members	Extended Members	Administrators
Orthopaedic Surgeon Radiologist Pathologist Medical Oncologist Radiation Oncologist	Spine Surgeon Paediatric Orthopaedic Surgeon Cardiothoracic Surgeon Plastic Surgeon Gynaecologist Physiotherapist Sarcoma Fellows Sarcoma Specialist Nurses	MDT Co-ordinator Pathway Co-ordinator

## Methods

The BST MDT at our service had 39 healthcare professionals across various specialities registered to attend the meeting in person on a weekly basis (Table 1). In 2019, an average of 51 cases (range 32–71) was discussed during every MDT meeting. Paediatrics, TYA, cardiothoracic surgery, spinal surgery, gynaecology, plastic surgery, and extremity surgery were the 7 sub-sections under which the cases were grouped. All cases were discussed between a musculoskeletal radiologist, an orthopaedic oncologist, clinical oncologists (radiation specialist and medical oncologist), a histopathologist and surgeons from the above respective sub-sections in order to generate a management plan for each patient. Spanning over 2 h, the MDT also routinely involved administrative staff, patient pathway coordinators, speciality nurses and clinical fellows.

Following the onset of the COVID-19 pandemic and subsequent enforcement of social distancing measures, from 23rd March 2020, the weekly MDT meetings at our Service were held via the virtual platform. The Trust installed a video-conferencing software whereby the administrator hosted the meeting and sent personalised links to each specialist member of the MDT. The video showing the essential imaging and patient data was screened at the MDT and the audio system involved discussed of all patient details by specialists facilitating to reach a diagnosis and plan management. Patients were discussed with their initials and patient anonymity was maintained at all times. The chairs of the meeting (musculoskeletal radiologist and orthopaedic oncologist) presented each case and rest of the participants dialled in via the videoconferencing software. In the eight MDTs held over the virtual platform, 393 cases were discussed (a mean of 49.1 cases/meeting) over the same two-hour period every week.

We formulated a questionnaire containing 24 questions pertaining to the functioning of MDTs prior to the pandemic and opinions on virtual MDTs following the start of the pandemic. The questionnaire was categorised into 4 sections. The first section included 7 questions pertaining to general information of the participant and their experience in MDTs involving cancer care. The second section involving 3 questions pertained to opinions on BST MDT functioning prior to the COVID-19 pandemic. The third section had 10 questions on experiences with the virtual MDTs during the pandemic. Participants were asked to compare virtual MDTs to conventional MDTs across seven criteria: depth of discussion, decision-making in diagnosis, change in plan of treatment, interaction with specialists, accessibility of relevant patient information, availability of expertise and time for discussion. The last section involving 4 questions focussed on opinions regarding the future scope of virtual MDTs based on the participants’ current experience.

The online survey was incorporated in Google Forms and circulated to all the 39 participants by email on 15th May 2020, following the completion of 8 virtual MDT meetings. After the first email, 22 responses were received. Following a reminder email, a further 14 responses were received.

## Results

We received 36 responses, corresponding to a response rate of 92.3% (36/39). Data was analysed and compiled using descriptive statistics. The average attendance rate of specialists was 94.8% in the 8 virtual MDTs.

The distribution of responses was as follows: orthopaedic surgeons (consultants and fellows) 22.2% (8/36), administrative support staff 16.6% (6/36), radiologists 13.8% (5/36), plastic surgeons 8.3% (3/36), clinical oncologists (medical oncology and radiation oncology) 8.3% (3/36), sarcoma specialist nurses 8.3% (3/36), pathologists 5.6% (2/36), spine surgeons 5.6% (2/36), paediatric orthopaedic surgeons 5.6% (2/36) and cardiothoracic surgeons 5.6% (2/36). Seventeen participants (47.2%) participated only in sarcoma MDT and 20 participants (55.6%) had previously participated in virtual MDTs prior to the pandemic.

### MDT functioning: pre COVID-19 pandemic

With regards to the opinion on conventional face-to-face MDTs, the norm prior to the pandemic, 47.2% (17/36) of the participants rated it to be ‘very good’, 47.2% (17/36) rated it to be ‘good’ followed by 5.6% (2/36) rating it to be ‘fair’.

### Comparison of virtual MDTs with conventional face-to-face MDTs

All participants reported that the COVID-19 pandemic had affected cancer care. Of the responses, 38.9% felt that it significantly affected patient care, 41.7% felt it affected care but not significantly, whereas 19.4% felt that it affected care only slightly. The majority of the participants (72.2%) approved the change from conventional face-to-face MDTs to virtual MDTs when it was implemented at the start of the pandemic.

72.2% were happy with depth of discussion happening through virtual MDTs and also felt that there was adequate interaction among specialists to discuss cases. Similarly, participants opined that the ‘change in treatment plan’ and ‘decision-making in diagnosis’ remained unaffected following the switch to the virtual platforms. About 86% felt the virtual platforms allowed them to have access to all relevant patient information (patient data and images) to chart out an adequate management plan. Nearly 89% felt that adequate availability of specialists to interact and plan treatment was available through the virtual MDT. Participants seemed divided over their overall opinion regarding their experience with virtual MDTs. While 36.1% were highly satisfied, 38.9% were moderately satisfied and 11.1% remained neutral; only 13.9% were dissatisfied with virtual MDTs.

### Opinion on future directions

Three-quarters (75%) of the participants were satisfied with the current experience and felt that the same standard of cancer care was provided as face-to-face MDTs. Following the present experience, 55.6% of the participants were happy to participate in MDTs only via the virtual platform whereas 33.3% wished to participate in them occasionally. In terms of the future, a significant majority (77.8%) felt that virtual MDTs are the future of modern cancer care and an even greater majority of 91.7% opined that the present pandemic experience of using videoconferencing platforms could pave the way towards global MDTs, whereby challenging cases could be discussed with specialists from across the globe.

## Discussion

**Fig. 1:**
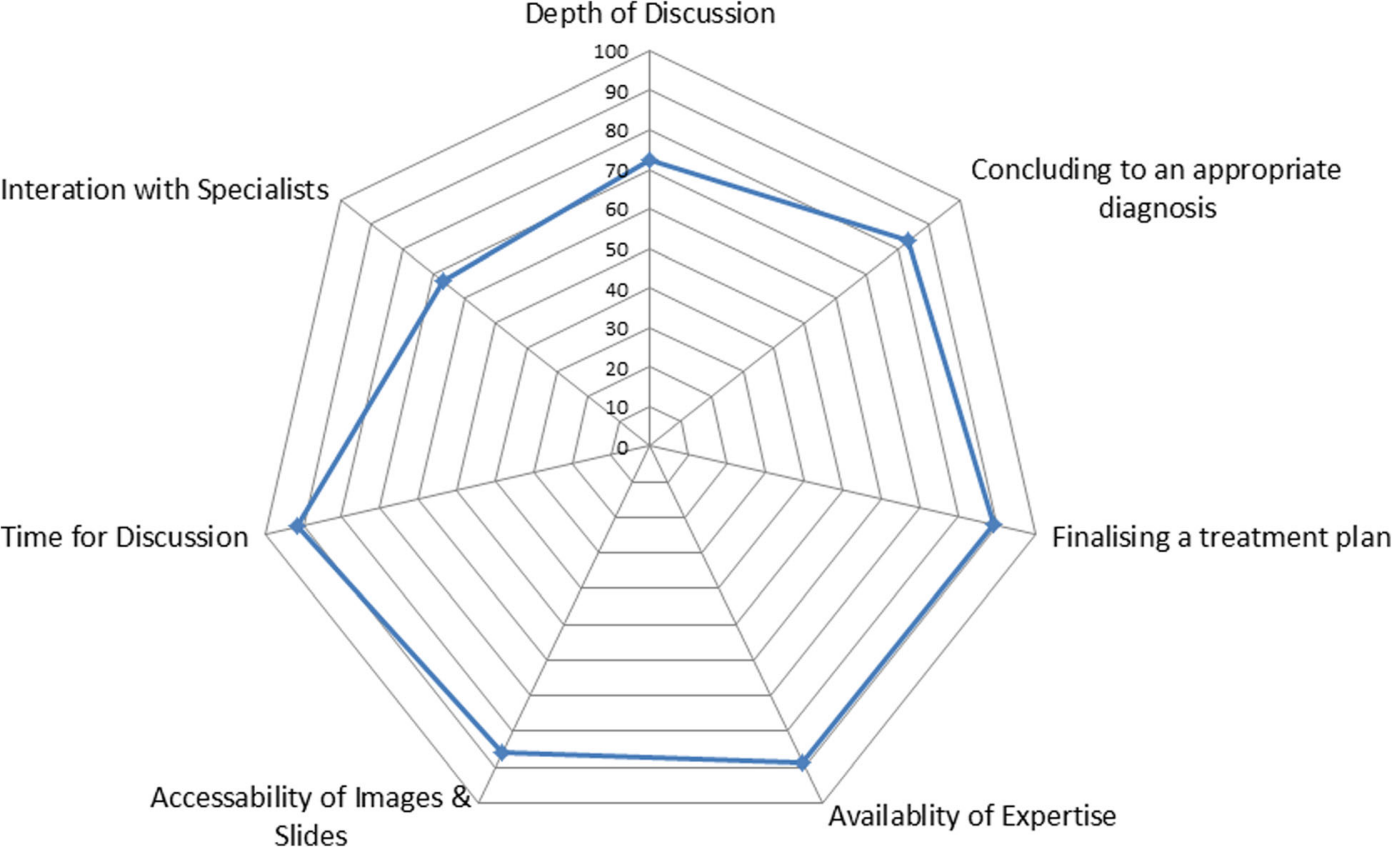
Radar plot depicting how important criteria evaluated on functioning of virtual MDTs compare to conventional face-to-face MDTs

Our survey results show that the vast majority (75%) of those surveyed reported that virtual MDTs provide the same standard of care as face-to-face MDTs and more than half (55.6%) would support the use of virtual MDTs following the end of the current pandemic and the ensuing return to normal work. Similarly, participants were satisfied with the depth of discussion taking place over virtual platforms and were able to access all relevant patient information to plan treatments (Fig. 1).

The role of an MDT in improving outcomes in cancer has been vastly reported across all cancers [1, 21, 22]. However, with regards to sarcoma, there is minimal literature on their efficacy. Analysis of outcomes from 26 referral centres in France involving 12,528 patients with sarcoma showed that patients discussed in a pre-therapeutic specialised MDT had a significantly better relapse-free survival [23]. The authors also stated that patients having their treatment planned in an MDT were more frequently managed with clinical practice guidelines. Improved consistency in decision-making, providing continuity of care and improved communication among healthcare professionals are the proven benefits of the MDT setting. Furthermore, MDT discussions also ensure appropriate treatment and early necessary interventions for patients.

In depth discussion of all patients by specialists following access to all relevant patient data is one of the key factors to widespread implementation of MDTs. The open nature of peer review at these meetings is beneficial in making the appropriate decision [24] and detailed discussion among specialists helps to achieve this goal. In our survey, 72.2% felt that this aspect was fulfilled in a virtual MDT. Two other key factors, ‘change in treatment plan’ and ‘decision making to achieve diagnosis’ also was found to be unaffected compared to face-to-face MDTs, as reported by the majority of the participants. A survey among participants who had been in virtual meetings involving head and neck cancers showed that 91.7% of them felt good access to patient data and adequate discussion over virtual platforms [25].

Another important result of our survey was that an overwhelming majority (88.9%) felt that adequate availability of specialists was possible on virtual platforms to discuss and plan treatments. This could be the major benefit of virtual MDTs as problems in attendance of specialists in conventional MDTs have been commonly reported [1]. A survey of 136 members attending breast cancer MDTs revealed that only 70% of clinical oncologists and 44% of medical oncologists attended the entire meeting [26]. Colorectal MDT attendance was variable with reported infrequencies among gastroenterologists and oncologists [27]. Most clinicians cited geographical location of meetings as the barrier as many specialists were working at various sites and also involved with several cancer services. In our sarcoma service, across 35 MDT meetings between June 2019 and February 2020, there was an attendance rate of 68.1%. With virtual MDTs, key members can still attend remotely to discuss their cases and need not travel to the location of the meeting. Apart from saving time travelling, it would also go a long way in improving the flexibility of clinicians’ work schedules.

In our survey, 56% claimed that they would be happy to attend MDTs only by a virtual platform in the future. The remaining 44% reported that difficulties in connectivity and technology were reasons for their reticence to attend virtual MDTs in the furture. A standardised approach applied to all teams working at multiple sites would be the solution to this problem. Two participants also reported that because of a lack of face-to-face interaction in the virtual MDTs, they were not able to adequately reinforce a point. Integrating appropriate decision making aids into artificial intelligence software could be the ideal way forward to provide effective functioning of virtual MDTs. Two participants who opined that they prefer face-to-face MDTs stated that the factor of ‘in-person communication’ was grossly missed on virtual MDTs. This factor helped in building stronger relationships, better team working and encouraged more robust conversations. This result was similar to the findings in a survey among participants of an MDT where virtual platforms had been implemented, in which 42% participants preferred ‘face-to-face’ MDTs for the same reasons [25].

Virtual MDTs have previously been proposed to manage cancer care. As discussed by Munro et al., virtual MDTs have certain advantages [16]. Increased participation of members as there is no geographical limitation, increased flexibility of scheduling and increased participation of members are a few. This is vital in the management of sarcomas given their rarity and given the necessity of experienced specialists required to manage them. Virtual MDTs may represent an ideal step forward in the MDT process to discuss all patients involving necessary specialists. Absence of even one key member could negatively affect decision-making and, therefore, patient outcomes. The concept of virtual MDTs has already been implemented in a few rare cancers requiring expert opinions. The UK National Ewing’s MDT was formed in 2011 and newly diagnosed cases of Ewing’s sarcoma were discussed through this virtual MDT and the treatment plans formulated [11, 28]. This implementation has been found to be effective as the best possible expertise is available for these complex, rare cancers and it also helps to maintain a national database for such cancers. Our results show that virtual MDTs could help expand institutional MDTs to regional MDTs and, thereby, to global MDTs where experts from around the globe gather across virtual platforms to offer expertise in complex MDTs. In our survey, an overwhelming majority of 91.7% felt that virtual MDTs would pave the way to expanding MDTs globally and 77.8% felt that virtual MDTs were synonymous with cancer care in future. Developing countries, where healthcare systems are less advanced, could employ these platforms and gather opinions from international centres of expertise, which could be a major advancement in cancer care.

Our study is not without limitations. These results are only from one centre and one specific cancer. Moreover, these results are only reflective of a small cohort. However, we believe that the respondents were of varying seniority across specialities, and we believe that our findings are generalizable to MDT functioning. Surveys carry an inherent risk of bias and often the response from clinicians may not reflect their true choice as they wish to provide a more academically acceptable answer rather than the most appropriate one. Another factor that may be influencing the opinions of participants is that these are only the early stages of virtual MDTs and with time, participants would be able to gauge their future role better. Only simple, descriptive statistics were performed and no detailed analysis was done as we believe that the results are quite well represented by simple statistics.

## Conclusion

An effective and well-attended MDT meeting with experienced specialists is essential, especially in the management of rare cancers such as sarcoma. Our study shows that the forced switch to virtual MDTs in sarcoma care following the unprecedented COVID-19 pandemic to be a viable and effective alternative to conventional face-to-face MDTs. Virtual MDTs could help expand the scope of expertise globally and could well be the future of cancer care.

## Supplementary Information

**Additional file 1.**

## Data Availability

The Questionnaire circulated among all participants has been attached as Supplementary Material. The datasets used and/or analysed during the current study are available from the corresponding author on reasonable request.

## Abbreviations

MDT: Multidisciplinary Team
BST: Bone and soft tissue tumour
