# Supplementary material for: Will virtual multidisciplinary team meetings become the norm for musculoskeletal oncology care following the COVID-19 pandemic? - experience from a tertiary sarcoma centre
Source: BMC Musculoskelet Disord. 2021 Jan 5;22:18. doi: 10.1186/s12891-020-03925-8 (PMC7784619; doi:10.1186/s12891-020-03925-8)
Supplement: Supplementary file 1 — Additional file 1. [file 12891_2020_3925_MOESM1_ESM.docx]

**Questionnaire:**

**General Information:**

1. Name: *(Optional)*
2. What is your Primary Speciality?
3. How many years have you been in this Speciality?
4. How many years have you been involved in Oncology?
5. From which year have you been participating in Bone & Soft Tissue (BST) Multi-disciplinary meeting (MDT) at Oxford?
6. Do you participate in any other MDT other than the Oxford BST MDT?

*Yes: If Yes, which are they?*

*No*

1. Have you participated in any virtual MDT before?

*Yes: If Yes, How many?*

*No*

**Opinion of MDT functioning: Pre-COVID-19:**

*Participants can answer these questions mentioning their opinions*

1. What was your opinion regarding the conventional face-to-face MDTs?

Poor

Fair

Good

Very Good

1. What are the most important advantages of a face-to-face MDT?
2. What are the disadvantages of a face-to-face MDT?

**Opinion of MDT functioning during Covid-19 pandemic: Virtual MDTs?**

1. Has the Covid-19 pandemic affected Cancer care?

*Yes, Significantly*

*Yes, but not significantly*

*Slightly*

*No, remains the same*

1. What was your opinion initially when the OUH Trust implemented virtual MDTs following the Covid-19 pandemic?

*Approved it*

*Neutral*

*Disapproved it*

**Depth of Discussion:**

1. Are you happy with the depth of discussion happening in the virtual MDTs compared with the conventional face-to-face MDTs?

*Yes*

*No*

**Coming to Diagnosis:**

1. Do you think decision making in concluding to a Diagnosis has been affected due to the switch to virtual MDTs?

*Yes*

*No*

1. **Change in Plan of management:**

Do you think there has been an increase in ‘change in treatment plan’ in vMDTs compared to conventional MDTs?

*Yes*

*No*

**Interaction:**

1. Were you able to interact adequately with other Specialists in the virtual MDT?

*Yes*

*No*

**Accessibility to relevant information:**

1. Were you able to access all relevant patient data (Images, Clinical details) in the virtual MDT?

*Yes*

*No*

**Availability of Expertise:**

1. Do you think adequate expertise of specialists was available in the decision making process?

*Yes*

*No*

**Time availability for Discussion:**

1. Do you think you have adequate time for discussion of cases in virtual MDTs?

*Yes*

*No*

1. Are you satisfied overall with virtual MDTs in BST?

*Highly satisfied*

*Moderately satisfied*

*Neutral*

*Dissatisfied*

*Highly dissatisfied*

**Future Directions:**

1. Do you think your current experience of virtual MDT will make you approve of virtual MDTs hereafter?

*Yes*

*No*

1. Will you be happy to attend the MDT by a virtual platform only in future?

*Yes*

*No*

*Maybe, occasionally*

1. Do you think virtual MDTs will be the future in treatment of Cancer care?

*Yes*

*No*

1. Do you think virtual MDTs will pave way to expanding MDTs globally (*seeking opinions on complex cases from specialist centres worldwide*)?

*Yes*

*No*

Any other comments with relation to the topic
